# The effects of telerehabilitation in adults with complex biventricular congenital heart conditions: protocol for a multi-centre, randomised controlled trial—CH-FIT

**DOI:** 10.1186/s13063-024-08019-7

**Published:** 2024-04-05

**Authors:** Gina Wood, Anna Scheer, Jelena Saundankar, Derek Tran, Rachael Cordina, Andrew Maiorana

**Affiliations:** 1 School of Allied Health, Curtin University, Perth, WA Australia; 2 School of Science and Technology, Faculty of Science, Agriculture, Business and Law, University of New England, Armidale, NSW Australia; 3 Perth Children’s Hospital, Cardiology, Nedlands Australia; 4 Sir Charles Gairdner Hospital, Cardiology, Nedlands Australia; 5 Department of Cardiology, Royal Prince Alfred Hospital, Sydney, NSW Australia; 6 Sydney Medical School, Central Clinical School, Faculty of Medicine and Health, The University of Sydney, Camperdown, NSW Australia; 7 Allied Health Department, Fiona Stanley Hospital, Perth, WA Australia

**Keywords:** Complex congenital heart anomalies, Combined exercise, Aerobic fitness, Telehealth, Neurocognitive function

## Abstract

**Background:**

Accumulated evidence suggests that exercise training exerts beneficial effects on people with congenital heart conditions. These findings are predominantly derived from small, single-centre exercise trials conducted in outpatient rehabilitation facilities. In recent years, the delivery of exercise interventions remotely has increased through digital communications technology (telerehabilitation). However, very little research to date has been conducted into the efficacy of telerehabilitation in people with a congenital heart condition.

**Aims:**

To evaluate the effects of a telehealth-delivered exercise intervention in people with a history of a surgical biventricular repair due to a congenital heart condition.

**Methods:**

One hundred eligible adolescent (≥ 16 years) and adult participants living with a complex biventricular congenital heart condition will be recruited from four Australian sites and randomised to either (1) a 16-week telehealth-delivered combined (aerobic and resistance) exercise training programme of moderate-to-vigorous intensity or (2) usual care (control group), in a 1:1 allocation, with an 8-month follow-up.

**Outcomes of interest:**

The primary outcome will be the change in aerobic capacity expressed as peak oxygen uptake (VO_2peak_). Secondary outcomes will include changes in vascular function, muscle oxygenation, metabolic profile, body composition and musculoskeletal fitness, neurohormonal activation, neurocognitive function, physical activity levels, dietary and nutritional status, and quality of life. Outcomes will be assessed at baseline, 16 weeks, and 12 months (to determine longer-term maintenance potential).

**Discussion:**

If found to be efficacious, telerehabilitation may be an alternative option for delivering exercise, improving health outcomes, and increasing accessibility to exercise programmes. Efficacy data is required to quantify the clinical significance of this delivery mode of exercise.

**Trial registration:**

ACTRN12622000050752

Trial registration date: 17 January 2022

Trial registration URL: https://www.anzctr.org.au/Trial/Registration/TrialReview.aspx?id=382635&showOriginal=true&isReview=true

Trial registry name: Australian and New Zealand Clinical Trials Registry

**Supplementary Information:**

The online version contains supplementary material available at 10.1186/s13063-024-08019-7.

## Introduction

Congenital heart conditions comprise any abnormality of the heart and central blood vessels present at birth. Elevated rates of morbidity and mortality, high medical resource consumption, and a dearth of efficacious treatments are associated with congenital heart physiologies of varying complexity [1].

Physical activity (PA) and structured exercise in sufficient amounts are recommended preventative and treatment methods for cardiovascular disease (CVD) [2, 3]. Historically, populations with congenital heart conditions have performed PA and exercise at volumes lower than recommended to achieve health benefits [4]. Adults with congenital heart pathologies of varying complexity exhibit impaired aerobic capacity (expressed as peak oxygen consumption; VO_2peak_) relative to age-predicted values, similar to that observed in patients with chronic heart failure [5]. This likely reflects a combination of pathophysiology and deconditioning exhibited by an abnormal ventilatory response to exercise and/or reduced exercise capacity, characteristics found to be associated with an increased 4-year risk of death or cardiac-related emergency hospital admission relative to patients with normal ventilatory responses and higher VO_2peak_ [6], and highlighting the prognostic significance of aerobic capacity. Moreover, an increase in VO_2peak_ of 6% is associated with a reduced mortality risk of 5% in chronic heart failure adults [7]. Although an association between improved aerobic capacity and clinical outcomes has yet to be established for congenital heart adults with varying pathologies, such an association is conceivable given (a) similarities in the pathophysiology between the two conditions and (b) that heart failure in cohorts with congenital heart conditions is the predominant cause of mortality and morbidity compared with chronic heart failure cohorts [8, 9] with > 90% of sudden cardiovascular deaths in adults with congenital heart conditions occurring at rest [10].

Behavioural, physical, and psychological factors contributing to decreased exercise and PA participation and capacity in congenital heart populations are manifold, including advice to avoid exercise [11, 12], reduced confidence to exercise [13], poor self-perception of exercise capacity [14], as well as low lean tissue/adiposity ratio [15], impaired muscle oxidative capacity and strength [16], impaired ventilatory and cardiopulmonary responses to exercise [17, 18], and decreased neurocognitive function [19–22]. However, the risk of adverse events during exercise is very low in people with congenital heart conditions. A recent systematic review reported no progression of symptoms, heart failure, or death, among the 403 participants reviewed [23].

Preliminary findings suggest that adults with congenital heart conditions tolerate and obtain benefit from appropriately prescribed exercise in gym-based programmes [24, 25], including improvement in aerobic exercise capacity and cardiac output [26–28], muscular function [29], and quality of life [30]. However, these studies have been limited by short duration (~ 12 weeks), design (non-randomised), and small sample size (< 50 participants).

To date, there are no randomised controlled trials testing the physiological and neurocognitive response to telerehabilitation treatment in adults with varying biventricular pathologies. Home-based cardiac rehabilitation is a viable treatment for clinically stable CVD patients [31]. Studies have shown telehealth-delivered exercise to be accessible and cost-effective in CVD patients [32, 33]. A possible means to reduce the financial impact associated with monitoring and rehabilitation of adults with chronic heart failure has been the judicious use of telehealth [34]: a recent study found potential to reduce costs associated with travel to attend medical appointments when replaced by telehealth delivery [35], and another study found equivalent care could be provided for lower cost [36]. The lack of larger, longer-duration high quality randomised controlled trials which redress lower rates of access and participation via innovative telerehabilitation and investigate the effect of exercise in adults with congenital heart pathologies prevents definitive exercise prescription as a treatment option for this cohort [37–39].

Biventricular congenital heart cohorts who undertake insufficient physical activity are at risk of impaired aerobic capacity and muscular strength, as well as potentially reduced neurocognitive function as documented in chronic heart failure populations, which may lead to further adverse outcomes and an increased burden on health systems. A strong justification is thus provided to investigate the effects of alternative modes of exercise prescription, such as structured telerehabilitation, to improve access to exercise training in people with congenital heart conditions. We hypothesise that, in this cohort, 16 weeks of a telehealth combined aerobic and resistance exercise training programme will demonstrate improvement in key physiological and psychological health parameters, compared with patients maintaining a usual and low level of activity.

## Methods

### Trial design

This will be a multi-site, two-armed, parallel-group, single-blind, superiority randomised controlled trial investigating the physiological and psychological effects of a telehealth-delivered 16-week aerobic and resistance exercise intervention in a biventricular congenital heart cohort, employing pre- and post-intervention measurements, with an 8-month follow-up.

### Trial setting and sites

Recruitment will occur at the Royal Prince Alfred Hospital, Royal Melbourne Hospital, Prince Charles Hospital, and Fiona Stanley Hospital in Australia. Potential recruits will be identified from the National Congenital Heart Disease Registry and trial site cardiologists. We include multiple sites in order to enhance participant enrolment and reach our target sample size.

### Participants

Medical records and telephone screening will be used to determine if potential recruits fulfil trial eligibility criteria. Treating or study physicians will confirm the suitability of potential recruits to enrol in the trial. Trial team members have consulted with various consumer organisations comprising individuals and their families with lived experience of congenital heart conditions while designing the trial, and this is expected to positively affect participant recruitment numbers. Additionally, information about the trial as well as participant information and recruitment material will be placed on institutional and study partner social media platforms and websites. A dedicated website page (https://www.hri.org.au/ch-fit-online-registrations) has been created where individuals can register their interest to participate and who can then be triaged to their nearest trial site.

### Inclusion criteria

Patients 16–55 years with biventricular congenital heart conditions of varying complexity, physiological stage B-C according to International Guidelines [40], and medically stable, who are > 6 months post-cardiac surgery as identified at the listed participating sites, and who may also present with conditions such as repaired tetralogy of Fallot and transposition of the great arteries, will be included. We have kept our inclusion criteria broad in order to achieve adequate participant enrolment.

### Exclusion criteria

Exclusion is (1) based on the last cardiologist’s assessment present in the recruit’s medical record and (2) determined at pre-intervention exercise testing. Exclusion criteria include simple lesion without sequelae such as a small ventricular septal defect, severe active enlargement of aorta, poorly controlled arrhythmias/blood pressure prior to or during exercise, non-optimal medical therapy and clinically unstable conditions in the preceding 3 months, severe outflow tract obstruction, severe valvular regurgitation, and uncontrolled systemic hypertension or decompensated heart failure (New York Heart Association (NYHA) class IV).

Further exclusion criteria include a physiological stage of D (severe hypoxaemia, systemic level pulmonary hypertension, Eisenmenger syndrome, refractory end-organ dysfunction) [40] as well as intellectual and/or physical disability preventing self-directed exercise, pregnancy or planned pregnancy (within 12 months), planned surgical intervention (within 2 years), self-reported volumes of PA or exercise sufficient to meet current minimum PA and exercise guidelines, and/or with no or an unreliable Internet connection. Our exclusion criteria aim to minimise the possibility of adverse or serious adverse events occurring, while still allowing for a broad recruitment strategy.

### Participant consent and withdrawal

Written, informed consent will be obtained at baseline visits, prior to assessment and randomisation, by site staff. Should an enrolled participant withdraw during the trial, they will be requested to complete a withdrawal form. Data collected from these participants will be retained for intention-to-treat analysis where consent is provided to retain data. Additional written, informed consent pertaining to outcomes requiring the collection of biological specimens via venesection and/or cannulation will also be obtained.

### Power analysis and statistical measures

A sample size of 100 is predicted to have ≥ 90% power (two-sided *α*: 5%) in estimating a VO_2peak_ difference of 10% between intervention and control (primary outcome), as suggested by preliminary findings [41]. We assume a 10% drop-out for a 16-week duration. Generalised linear mixed models with random subject and site effects (to control for variation between individuals and the clustering effects of site) and group-time interactions will be used to estimate the between and within group pre- and post-intervention changes in the primary outcome, controlling for covariates that may influence the outcome. Results will be summarised using descriptive and inferential statistics, with relevant *P* values (significance < 0.05). Sensitivity analysis of per-protocol vs intention-to-treat will be conducted. We will handle missing data by imputation using the closest match method. i.e. substitute a same time-point value from a participant with the closest value for the measured variable at other time points [42].

### Randomisation and concealment

**Fig. 1:**
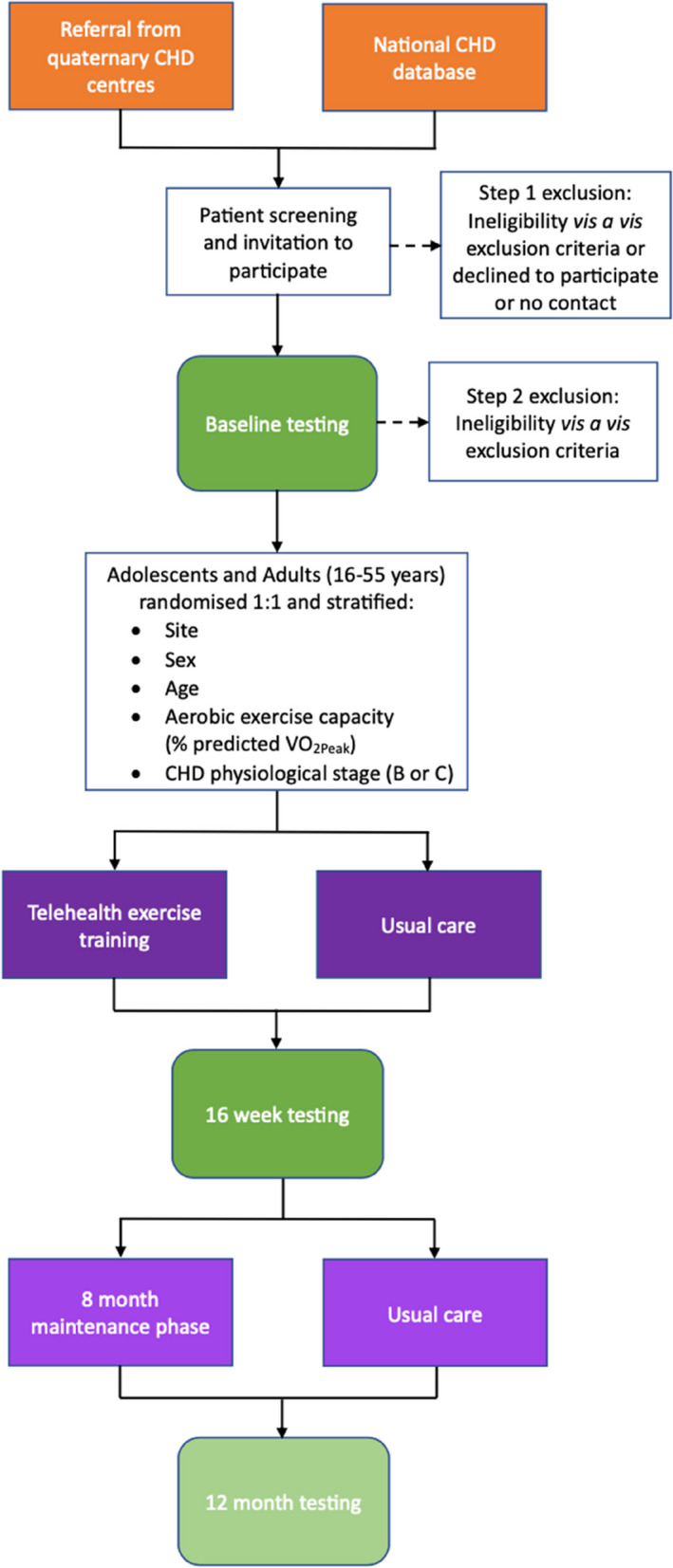
The Congenital Heart Fitness Intervention Trial (CH-FIT) study design flow chart

Computer-generated randomisation will be stratified on a 1:1 allocation by site, baseline aerobic exercise capacity (% predicted VO_2peak_), physiological stage (B or C), age, and sex (female, male), per Fig. 1. An independent statistician/staff member will employ randomisation by minimisation to allocate participants. Where possible, site assessors will be blinded to participant group allocation and identification. Participants’ data and identification will be stored centrally (REDCap™) and accessible only by a trial coordinator (uninvolved in participant enrolment, randomisation, or assessment). Unblinding is expected to occur in the event of a serious adverse or adverse event. Participants are not blinded owing to the nature of the intervention (exercise).

### Flow of participants (recruitment to completion) and schedule

Figure 1 presents the study design flow chart of participants, and Table 1 lists the schedule of recruitment, consent, enrolment, assessments, randomisation, intervention, and follow-up periods. The recruitment period is expected to last for approximately 30 months, with an expected monthly rate of recruitment commensurate with each site’s participant allocation (generally 3–4 participants per month across all sites). In addition, sites will over-recruit by up to 10% in order to achieve sufficient participant enrolment to attain the required adequately powered sample size.

**Table 1:**
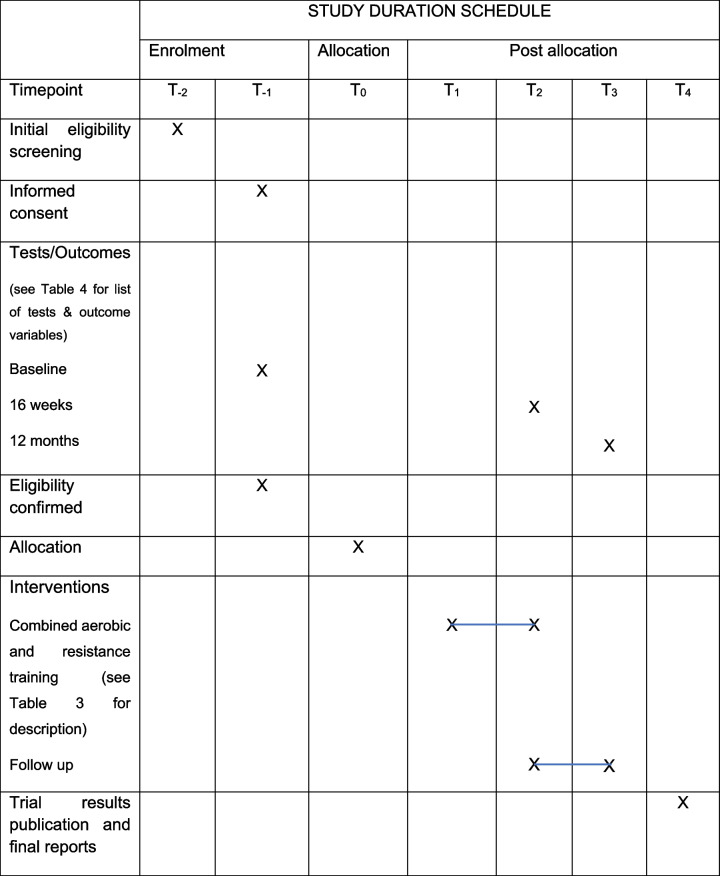
The Congenital Heart Fitness Intervention Trial (CH-FIT) schedule of visits (Tables 3 and 4 )

For each trial site, the target sample size is summarised in Table 2.

**Table 2:** Participant numbers (intervention and control) per trial site

Site name	Intervention telehealth (*n*)	Control (*n*)	Total (*n*)
Royal Prince Alfred Hospital (RPAH)	17	18	**35**
Royal Melbourne Hospital (RMH)	13	12	**25**
Prince Charles Hospital (PCH)	8	7	**15**
Fiona Stanley Hospital (FSH)	12	13	**25**
**Total (** ***n*** **)**	**50**	**50**	**100**

## Intervention

The telerehabilitation intervention comprises unsupervised moderate-to-vigorous intensity aerobic training combined with telehealth-delivered supervised resistance training, summarised in Table 3 (either one continuous aerobic and resistance session or two separate sessions according to participant preference).

**Table 3:** Excluding warm-up and cool down of ~ 10 min (total time is approximately 30–40 min per individual session)

Weeks (sessions)	Intensity category	Objective intensity	Subjective intensity
**Aerobic exercise training progression**			
1–2 (6)	Moderate	40–50% HRR	4 OMNI-RES Scale
3–6 (12)	Moderate	50–60% HRR	5 OMNI-RES Scale
7–10 (12)	Vigorous	60–70% HRR	6 OMNI-RES Scale
11–16 (18)	Vigorous	70–80% HRR	7 OMNI-RES Scale
**Resistance exercise training progression**			
1–2 (6)	Moderate (moderate load)	60% 1RM (3 sets, 8–12 repetitions)	4–5 OMNI-RES Scale
3–16 (42)	Vigorous (moderate-to-high-load)	70% 1RM (3 sets, 8–12 repetitions)	6–7 OMNI-RES Scale

### Aerobic training protocol

Participants will receive a heart rate (HR) monitor transmitting real-time exercise intensity data to an electronic device enabled with the Polar Flow Mobile App™. Additionally, participants will be asked to record per-session exercise duration and per-session average and peak HR as well as rate of perceived exertion (RPE) according to the OMNI-RES scale. Participants will be instructed by qualified exercise professionals on how to achieve exercise intensity commensurate with progressive target levels. Self-selected aerobic exercise modalities may include either indoor or outdoor walking/jogging/running, dancing, cycling, skipping, stepping, or any other form of continuous aerobic exercise sustained within the prescribed HR reserve (HRR) or RPE range (see Table 3) to be performed for 30 min, including 5 min each of warm up and cool down, three times per week. Participants will receive a complimentary gym membership to facilitate adherence to unsupervised aerobic exercise training.

### Resistance training protocol

Participants will be provided with a resistance exercise device (Gymstick™): a cylindrical fibre-glass rod of 1300 mm length and 30 mm diameter. At each of the stick, interchangeable flexible rubber tubes of varying resistance are attached with the other end of the tube looped at either of feet or hands. Qualified exercise professionals will instruct participants on the correct use of the device and how to achieve desired intensity on a progressive basis as well as supervise small group resistance training sessions via Zoom™. Training sessions will comprise three sets of 8–12 repetitions of 6 resistance exercises at targeted and progressing percentage intensities of one repetition maximum (1RM) three times per week (see Table 3) for approximately 40 min per session including 5 min each of warm up and cool down. Participants will be educated on appropriate breathing during each phase of the resistance exercises and shown how to avoid the Valsalva manoeuvre. If onset of symptoms, fatigue, or inability to complete a set of resistance exercises at the lowest resistance level or bodyweight occurs, longer intra-set and inter-set rest periods will be permitted.

###  Combined exercise training protocol

The combined exercise training protocol is presented in Table 3.

### Adherence, compliance and activity monitoring

Adherence and compliance to exercise training will be monitored digitally (HR device, attendance at Zoom™ sessions) as well as via analog (training logs). Attending 80% of the prescribed sessions—with attendance to > 70% of sessions in the 4 weeks preceding the follow-up assessment visit—is considered adherence to the intervention. Attendance of 20–79% of the prescribed sessions are semi-adherent participants. Non-adherent participants are those that attend < 20% of the prescribed sessions. Compliance is defined as those participants who have worked for > 50% of the session at or above the target intensity. Compliance is reported for aerobic and resistance training per session. Sessional compliance requires meeting the target training criteria for aerobic and resistance training. During the intervention and the 8-month follow-up period, intervention participants are contacted monthly to monitor adverse events and change in PA levels.

## Comparator

### Controls

Participants allocated to the control group will continue with routine clinical care (monitoring and/or medication) and undergo the same assessments, conducted at baseline, 16 weeks, and 12 months, as those participants allocated to the intervention group. Physical activity levels in control group participants will be monitored at pre- and post-intervention. In order to avoid confounding effects, control group participants are neither reminded of nor provided with a means to track PA levels during the intervention period but will be instructed to continue with usual PA and exercise levels. During the intervention and the 8-month follow-up period, control group participants are contacted monthly to monitor adverse events and change in PA levels. Control group participants will be presented with the opportunity to join a later 16-week telerehabilitation programme comprising the same intervention.

## Outcomes

Assessments will be performed at baseline, 16 weeks, and at 12 months to obtain the data required for evaluating the trial hypotheses and achieving trial objectives, as per Table 4.

**Table 4:** Tests and relevant parameters measured necessary to achieve trial objectives

**Test**	**Parameters studied (primary outcome, all sites)**
Exercise test on cycle ergometer (cardiopulmonary exercise test (CPET)) using a ramp protocol	Cardiorespiratory fitness (VO_2PEAK_)
**Test**	**Parameters studied (secondary outcomes** (selected sites and/or ancillary studies))
Exercise test on cycle ergometer (cardiopulmonary exercise test (CPET)) using a ramp protocol	Pre-exercise spirometry, HR and rhythm, oxygen saturation, blood pressure, and ventilatory response to exercise
Strength testing	1-repetition maximum (1RM) for leg press, seated row and chest press
Near-infrared spectroscopy (NIRS)	Skeletal muscle oxidative capacity
Flow-mediated dilation	Brachial artery diameter and assessment of endothelial function
Dual-energy X-ray absorptiometry (DEXA)	Lean appendicular muscle mass, fat mass, bone mineral content, and bone density
Accelerometer (ActiGraph GT9X Link device) for participants	7-day measurement (minimum 3 days each 8 h) for each group of parameters pertinent to physical activity, activity intensity, sedentary time, and sleep and sleep quality
Automated Self-Administered Recall System (ASA24), Gastrointestinal Symptom Rating Scale (GSRS), and Subjective Global Assessment (SGA)	Dietary intake history compiled by recalling type and quantity of nutrition ingested Gut function assessed by self-evaluation of the following five symptom clusters: reflux, abdominal pain, indigestion, diarrhoea, and constipation State of nourishment (well-, mal-) clinically assessed using functional capacity, solids/fluids intake, metabolic requirement, and physical examination
Self-administered digital Cogstate assessment—tests include detection, identification, one card learning, one back test, Groton maze chase, Groton maze learning, international shopping list, and social emotion cognition	Cognitive function assessed for the following domains: p sychomotor function, attention, visual learning/memory, working memory, processing speed, executive function, verbal learning/memory, and social emotional cognition
Duke Activity Status Index, Self-assessed NYHA functional classification	Self-assessed functional capacity determined by ability to participate in daily activities of living such as self-care, housework, gardening, and recreational physical activities, and presence/severity of symptoms (e.g. fatigue, palpitations, dyspnoea) accompanying each activity
Fatigue Severity Scale	Self-assessed levels of fatigue for the past 7 days encompassing causes of fatigue, problems caused by fatigue, and effect of fatigue on daily activity types and levels
EQ-5D [43]	Self-assessed health-related quality of life encompassing levels of mobility, self-care, physical activities, pain/discomfort, and anxiety/depression
International physical activity questionnaire and exercise self-efficacy questionnaire [44]	Self-reported habitual physical activity and self-efficacy levels using volume of time spent in various physical activities
Laboratory assessments via blood collection to capture relevant biomarkers (not all sites)	1. Full blood count (FBC) 2. N-terminal pro hormone B-type natriuretic peptide (NT-proBNP) 3. Highly sensitive C-reactive protein (hsCRP) 4. Comprehensive metabolic panel including alanine aminotransferase (ALT), aspartate transaminase (AST), bilirubin, urea, creatinine, calcium, magnesium, phosphates, electrolytes, and fasting blood glucose levels (FBG) 5. Fasting insulin 6. Prealbumin 7. Fasting lipid profile plus apolipoprotein A1 and B100 8. Multi-omic analyses (plasma)

###  Physiological and psychological assessment measurements

The physiological and psychological assessment measurements are presented in Table 4.

## Trial data collection, confidentiality, storage, and archival

Data will be collected in accordance with CONSORT and the supplementary CERT reporting guidelines. Data will be saved in the password-protected online REDCap™ database. Participants will be allocated a unique REDCap™ identification code. Access to identifiable data will only be permitted for designated researchers, the permissions for which are controlled by the trial coordinator. Source documents will be stored in individual patient files as hard copies and encrypted as secure electronic patient files with restricted access to designated researchers. A research data management plan has been developed covering the confidentiality, storage, and archiving plans of study data, which complies with the relevant statutory requirements.

## Adverse events/serious adverse events

Adverse events (AE) such as the occurrence of an adverse medical symptom and serious AEs such as death, hospitalisation, or progression of symptoms according NYHA classification will be recorded in REDCap™ and reviewed every 12 weeks from commencement of recruitment. Enrolled participants will be provided with a procedure to follow in the event of the onset of adverse medical symptoms during either an unsupervised aerobic or supervised telehealth resistance training session. Exercise professionals conducting the telehealth resistance training sessions will be trained in the appropriate process in the event of either an AE or a serious AE. A data safety and monitoring board comprising members independent of the trial with no competing interests and including clinical experts and a biostatistician will be created to ensure the process to be followed by recruits and/or staff during the intervention and in case of the occurrence of an AE or serious AE as well as to recommend cessation of the trial to lead investigators if necessary (AE or serious AE). Further roles and responsibilities of the board will be outlined in its charter, and the board will meet every 3 months to review trial progress, conduct, and AEs/serious AEs. Reports on trial progress will be filed with participating site governance bodies annually as well as to the trial sponsor and funding body.

## Post-trial care and compensation

Following the completion of the 12-month follow-up, it is expected that enrolled participants will continue with usual care as well as an augmented exercise programme devised in conjunction with their usual cardiologist and allied health practitioner. If a participant suffers any injuries or complications as a result of the research project, they will be advised to contact the trial team, who will assist with arranging appropriate medical treatment.

In addition, patients may have a right to take legal action to obtain compensation for any injuries or complications resulting from the study. Compensation may be available if their injury or complication is sufficiently serious and is caused by unsafe drugs or equipment or by the negligence of one of the parties involved in the study (for example, the researcher, the hospital, or the treating doctor). Patients do not relinquish any legal rights to compensation by participating in this trial.

## Discussion

We have developed a protocol for an adequately powered, multi-centre, 16-week randomised controlled trial with an 8-month follow-up period investigating the effects of telerehabilitation in adults (≤ 55 years) and adolescents (≥ 16 years) diagnosed with biventricular congenital heart conditions. To the best of our knowledge, this will be the first trial delivering such an intervention via a novel platform (telehealth) to this cohort. We have adopted a pragmatic approach in our inclusion and exclusion criteria to enhance recruitment, while simultaneously limiting the potential for adverse events to occur in a group with cardiac physiologies of varying complexity.

A strength of our protocol is the objective measurement of key physiological variables during exercise training as well as objective confirmation of compliance and adherence to the exercise intervention. The monitors used during exercise provide real-time data, and detailed feedback is available from the Polar Flow Mobile App™. In addition, our trial intervention and objectives have been developed in consultation with consumer organisations such as HeartKids and the Australian and New Zealand Fontan Registry, comprising individuals and their families with lived experience of congenital heart conditions as well as people with significant expertise in the management and support of those affected by congenital heart conditions. This is expected to assist in recruitment as well as maintaining trial compliance and adherence.

A possible limitation of our study could be the multi-centre facet, which has the potential to introduce discrepancies in accuracy and reliability of collected data and measurements of physiological and psychological parameters. We address this limitation in two ways: (1) we use self-administered and validated online tests, which reduces both assessor bias and non-reproducibility of results, and (2) we have developed standard quality assurance and control procedures for use of required equipment in line with manufacturer’s specifications. All staff will have received standardised training prior to interacting with participants. We are thus able to leverage a robust geographical diversity.

The number of aging adults with biventricular congenital heart conditions and historically low volumes of PA and exercise is rising. It is hypothesised that telerehabilitation will improve multiple health outcomes, such as increasing VO_2peak_, which is a strong prognostic indicator in people with cardiac conditions. Exercise-induced increases in muscular strength, bone density, quality of life, and neurocognitive function are expected to provide a broad range of tangible benefits. Moreover, this trial will evaluate these outcomes in the context of a novel method of intervention delivery, encouraging accessibility to and participation in exercise in a group with generally depressed exercise volumes. In addition to updating the Trial Registry with our findings, we expect that the results of our trial will be disseminated not only through peer-reviewed publications but also through the members of consumer groups consulted prior to the trial commencement, via their subscriptions to social media and other digital channels as well as consumer organisations such as HeartKids via their websites, workshops, and support programmes, and that these consumer organisations will advocate for the incorporation of our findings in policy and practice.

## Trial status

Recruitment commenced on 15 June 22. Recruitment is expected to be completed by end December 2024. The current trial protocol for all study arms submitted and approved is Version 9, date of approval 08 March 2024. Documented changes include the addition of study team members for all study arms and minor administrative amendments required by state legislating bodies responsible for each participating site.

### Supplementary Information

**Supplementary Material 1.**

**Supplementary Material 2.**

**Supplementary Material 3.**

## Data Availability

The de-identified datasets that will be generated and analysed from the trial, as well as the statistical code, may be made available from the corresponding author on reasonable request, as will be the full protocol, after completion of the trial.

## Declarations

### Trial protocol version

Version 9, approved 08 March 2024.

## References

[1] Mylotte D, Pilote L, Ionescu-Ittu R (2014). Specialized adult congenital heart disease care: the impact of policy on mortality. Circulation.

[2] Smith SC, Jackson R, Pearson TA (2004). Principles for national and regional guidelines on cardiovascular disease prevention. Circulation.

[3] Naci H, Ioannidis JPA (2015). Comparative effectiveness of exercise and drug interventions on mortality outcomes: meta-epidemiological study. Br J Sports Med.

[4] Pinto NM, Marino BS, Wernovsky G (2007). Obesity is a common comorbidity in children with congenital and acquired heart disease. Pediatrics.

[5] Diller G-P, Dimopoulos K, Okonko D (2005). Exercise intolerance in adult congenital heart disease. Circulation.

[6] Giardini A, Hager A, Lammers AE (2009). Ventilatory efficiency and aerobic capacity predict event-free survival in adults with atrial repair for complete transposition of the great arteries. J Am Coll Cardiol.

[7] Swank AM, Horton J, Fleg JL (2012). Modest increase in peak VO2 is related to better clinical outcomes in chronic heart failure patients: results from heart failure and a controlled trial to investigate outcomes of exercise training. Circ Heart Fail.

[8] Shekhar S, Agrawal A, Pampori A (2022). Mortality in adult congenital heart disease: analysis of outcomes and risk stratification. J Cardiothorac Vasc Anesth.

[9] Diller G-P, Kempny A, Alonso-Gonzalez R (2015). Survival prospects and circumstances of death in contemporary adult congenital heart disease patients under follow-up at a large tertiary centre. Circulation.

[10] Zomer AC, Vaartjes I, Uiterwaal CSPM (2012). Circumstances of death in adult congenital heart disease. Int J Cardiol.

[11] Swan L, Hillis WS (2000). Exercise prescription in adults with congenital heart disease: a long way to go. Heart.

[12] Reybrouck T, Mertens L (2005). Physical performance and physical activity in grown-up congenital heart disease. Eur J Cardiovasc Prev Rehabil.

[13] Sandberg C, Thilén U, Wadell K (2015). Adults with complex congenital heart disease have impaired skeletal muscle function and reduced confidence in performing exercise training. Eur J Prev Cardiol.

[14] Gratz A, Hess J, Hager A (2008). Self-estimated physical functioning poorly predicts actual exercise capacity in adolescents and adults with congenital heart disease. Eur Heart J.

[15] Sandberg C, Johansson K, Christersson C (2019). Sarcopenia is common in adults with complex congenital heart disease. Int J Cardiol.

[16] Kröönström LA, Johansson L, Zetterström A-K (2014). Muscle function in adults with congenital heart disease. Int J Cardiol.

[17] Bassareo PP, Saba L, Solla P (2014). Factors influencing adaptation and performance at physical exercise in complex congenital heart diseases after surgical repair. Biomed Res Int.

[18] Dimopoulos K, Diller G-P, Piepoli MF (2006). Exercise intolerance in adults with congenital heart disease. Cardiol Clin.

[19] Verrall CE, Yang JYM, Chen J (2021). Neurocognitive dysfunction and smaller brain volumes in adolescents and adults with a fontan circulation. Circulation.

[20] Perrotta ML, Saha P, Zawadski R (2020). Adults with mild-to-moderate congenital heart disease demonstrate measurable neurocognitive deficits. Am Heart Assoc.

[21] Cohen S, Earing MG (2018). Neurocognitive impairment and its long-term impact on adults with congenital heart disease. Prog Cardiovasc Dis.

[22] Ilardi D, Ono KE, McCartney R, Book W, Stringer AY (2017). Neurocognitive functioning in adults with congenital heart disease. Congenit Heart Dis.

[23] Li X, Chen N, Zhou X (2019). Exercise training in adults with congenital heart disease: a systematic review and meta-analysis. J Cardiopulm Rehabil Prev.

[24] Tran D, Maiorana A, Ayer J (2020). Recommendations for exercise in adolescents and adults with congenital heart disease. Prog Cardiovasc Dis.

[25] Chaix M-A, Marcotte F, Dore A (2016). Risks and benefits of exercise training in adults with congenital heart disease. Can J Cardiol.

[26] Ubeda Tikkanen A, Opotowsky AR, Bhatt AB (2013). Physical activity is associated with improved aerobic exercise capacity over time in adults with congenital heart disease. Int J Cardiol.

[27] Cordina RL, O'Meagher S, Karmali A (2013). Resistance training improves cardiac output, exercise capacity and tolerance to positive airway pressure in Fontan physiology. Int J Cardiol.

[28] Sheng P, Feinberg JL, Bostrom JA (2022). Adherence and exercise capacity improvements of patients with adult congenital heart disease participating in cardiac rehabilitation. J Am Heart Assoc.

[29] Ntelios D, Giannakoulas G, Dimopoulos K (2019). Strength training in congenital heart disease: a way to boost respiratory function?. Eur J Prev Cardiol.

[30] Sandberg C, Engström KG, Dellborg M (2015). The level of physical exercise is associated with self-reported health status (EQ-5D) in adults with congenital heart disease. Eur J Prev Cardiol.

[31] Thomas R, Beatty A, Beckie T (2019). Home-based cardiac rehabilitation. J Am Coll Cardiol.

[32] Maddison R, Rawstorn JC, Stewart RAH (2019). Effects and costs of real-time cardiac telerehabilitation: randomised controlled non-inferiority trial. Heart.

[33] Brouwers RWM, van der Poort EKJ, Kemps HMC (2021). Cost-effectiveness of cardiac telerehabilitation with relapse prevention for the treatment of patients with coronary artery disease in the Netherlands. JAMA Netw Open.

[34] Grustam AS, Severens JL, De Massari D (2018). Cost-effectiveness analysis in telehealth: a comparison between home telemonitoring, nurse telephone support, and usual care in chronic heart failure management. Value in Health.

[35] Snoswell CL, Taylor ML, Comans TA (2020). Determining if telehealth can reduce health system costs: scoping review. J Med Internet Res.

[36] Hwang R, Morris NR, Mandrusiak A (2019). Cost-utility analysis of home-based telerehabilitation compared with centre-based rehabilitation in patients with heart failure. Heart Lung Circ.

[37] Williams CA, Wadey C, Pieles G (2020). Physical activity interventions for people with congenital heart disease. Cochrane Database Syst Rev.

[38] Longmuir PE, Brothers JA, Ferranti SD (2013). Promotion of physical activity for children and adults with congenital heart disease. Circulation.

[39] Xu C, Su X, Ma S (2020). Effects of exercise training in postoperative patients with congenital heart disease: a systematic review and meta-analysis of randomized controlled trials. J Am Heart Assoc.

[40] Stout KK, Daniels CJ, Aboulhosn JA (2019). 2018 AHA/ACC guideline for the management of adults with congenital heart disease: executive summary: a report of the American College of Cardiology/American Heart Association Task Force on clinical practice guidelines. Circulation.

[41] Cordina R, O’Meagher S, Karmali A (2013). Resistance training improves cardiac output, exercise capacity and tolerance to positive airway pressure in Fontan physiology. Int J Cardiol.

[42] Elliott P, Hawthorne G (2005). Imputing missing repeated measures data: how should we proceed?. AUST N Z J Psych.

[43] Brooks R, Boye KS, Slaap B (2020). EQ-5D: a plea for accurate nomenclature. J Patient-Rep Outcomes.

[44] Resnick B, Jenkins LS (2000). Testing the reliability and validity of the self-efficacy for exercise scale. Nurs Res.

