# Supplementary material for: The effects of telerehabilitation in adults with complex biventricular congenital heart conditions: protocol for a multi-centre, randomised controlled trial—CH-FIT
Source: Trials. 2024 Apr 5;25:239. doi: 10.1186/s13063-024-08019-7 (PMC10998335; doi:10.1186/s13063-024-08019-7)
Supplement: Supplementary file 2 — Supplementary Material 2. [file 13063_2024_8019_MOESM2_ESM.docx]

**[LETTERHEAD OF RESEARCHER’S INSTITUTION]**


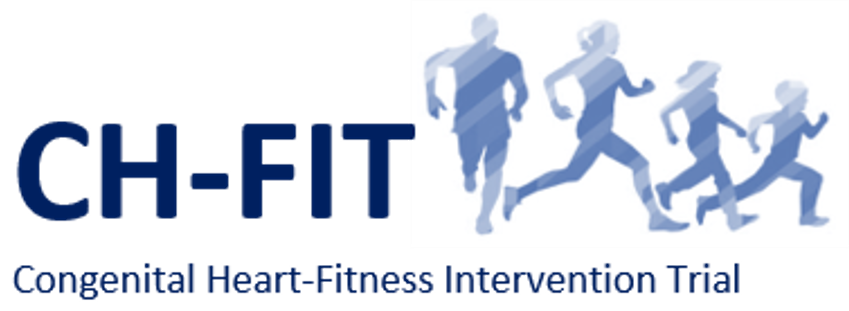


**Optimising Exercise Prescription and Delivery in Congenital Heart Disease – The Congenital Heart Fitness Intervention Trial: CH-FIT**

INFORMATION FOR PARTICIPANTS

**Principal Investigators**

[NAMES OF PRINCIPAL INVESTIGATORS]

Associate Investigators

[NAMES OF ASSOCIATE INVESTIGATORS]

# Introduction and purpose of THE study

You are invited to take part in a research study investigating the effects of exercise training in people living with congenital heart disease. You have been invited because you have congenital heart disease.

Major advances in medicine and surgical techniques have dramatically improved survival. However, people living with congenital heart disease still experience long-term health complications. An important marker of health in people with congenital heart disease is exercise capacity. Better exercise capacity is related to better health outcomes and heart function. Importantly, exercise capacity is something we can improve with exercise training. Consistent exercise training has also been shown to improve heart function, body composition, vascular function, quality of life and decreased occurrences of arrhythmias in people with congenital heart disease.

Traditional models of exercise training such as cardiac rehabilitation may not be suitable or optimal for people with congenital heart disease. Although exercise training is safe in people with congenital heart disease, the best type and amount of exercise required for improvements in health outcomes is still unknown. Your participation will help to provide us with valuable information to optimise exercise prescriptions for people with congenital heart disease. The Congenital Heart Fitness Intervention Trial (CH-FIT) will be the first study of its kind in the world to apply resistance and aerobic exercise training online to improve health outcomes for adolescents and adults with congenital heart disease.

Our primary aim with this research is to investigate the impact of a moderate-to-vigorous telehealth exercise training program in people with congenital heart disease. We will achieve this by evaluating changes in diet, body composition (i.e., bone, fat, muscle mass), strength, lung function, physical activity levels, vascular function, quality of life (QOL), heart function, and cognitive function.

Please read this Participation Information Sheet carefully and ask any questions about the information in this document. This Participant Information Sheet contains details about the study and explains the tests involved so that you can make an informed decision about your participation. Participation in this study is voluntary. If you do not want to take part, you do not have to. Your decision to participate or not will not affect your quality of care.

You are free to withdraw from this study at any time if you wish. If you choose to withdraw from this study, this will not impact your care or relationship with the hospital or university. If you decide to enrol in this study, you will be asked to sign the consent form. By signing the consent form, you are telling us that you:

- Understand what you have read.
- Consent to take part in the study.
- Consent to the tests described.
- Consent to the use of your personal and health information as described.

You will be given a copy of this Participant Information Sheet to keep.

# What does participation in the study involve?

If you consent to participate in this study, you will be asked to attend the hospital and/or university to complete a variety of study investigations at the start of the study (baseline), after 4-months, and after 12-months. Each study investigation visit will take a full day, and some people may be asked to attend a second half-day of testing. If you are eligible after the first study investigation visit (baseline), you will be randomly allocated to an exercise intervention group or a waitlist control group.

## Study Investigations

You will be asked to attend the hospital or university at least three times to complete some study investigations. Some of the study investigations will require some preparation before your visit. Please see the **“How do I prepare for my testing visit?”** sheet for more information. Study staff will also inform you of any preparation required before your study investigation visit. During your follow-up study investigation visits, we ask you to refrain from informing your assessor of the group you have been allocated to. The different types of tests are described in the below table.

| **Study investigation** | **What will the study investigations involve?** |
| --- | --- |
| **Height, weight, resting haemodynamics, and basic body composition measurements** | A research staff member will measure your height, weight, blood pressure, and resting heart rate. Your basic body composition will also be measured using bioelectrical impedance analysis, which requires you to stand on a scale-like device and hold onto some handles for a few seconds. |
| **Cardiopulmonary exercise test** | This test will assess your exercise capacity and exercise response. It involves an 8-15 minute maximal exercise test on a stationary bike while you breathe through a “scuba-like” mouthpiece or face mask. To monitor your heart rhythm and exercise response, a blood pressure cuff, ECG, and oximeter may also be attached to you. This test is part of your routine clinical care. |
| **Strength testing** | To measure your muscular fitness, you will be shown how to do specific strength exercises. Then you will be asked to perform some maximal lifting, pushing, and pulling tests in the hospital or university gym using resistance machines to determine the maximum load you can lift once. You will also be asked to push a weight as many times as you can for a specific exercise to assess your muscular endurance. |
| **Detailed body composition scan** | For a more detailed assessment of your body composition (i.e., amount of muscle, bone and fat), a DXA scan will also be performed. The scan requires you to lie still on a scanning table for approximately 10 minutes. |
| **Computer-based cognitive assessment (Cogstate)** | You will be asked to complete a series of computer-based cognitive tasks. This will involve completing a series of tasks assessing your ability to identify and detect objects/stimulus and will involve card and maze learning tasks. |
| **Questionnaires** | You will be asked to answer a series of questionnaires about your nutrition and gut function, pubertal status, functional capacity, fatigue, physical activity levels, exercise-self efficacy, quality of life, mood and health. Each questionnaire should take approximately 5-15 minutes to complete. You will be asked to either complete this before your assessment date online or throughout the testing day. |
| **Blood test** | For the blood test, you will be asked to fast overnight (8-12 hours). We will collect ~10-32mL of blood to measure specific proteins that reflect heart function, metabolism, and other markers of organ function. This test is part of your routine care. Your blood may also be stored for future analysis. |
| **Physical activity level** | To measure your daily physical activity levels, you will be asked to wear a physical activity watch on your non-dominant wrist for seven days. After 7 days, you will be asked to send the watch back to the study team using a prepaid satchel that will be provided to you. |
| **Gut microbiome and metabolites** | To understand your gut health, the research study team will provide you with a stool collection kit and a return postage satchel. You and a household member will be asked to collect a small amount of stool using the kits and send it back to the study team within 2 days. |
| **Detailed nutritional and dietary assessment** | You may also undergo a detailed dietary assessment. During the dietary assessment, a research staff member or qualified health professional will ask you about your diet history and perform a quick non-invasive physical assessment to assess your nutritional status. You will also be asked to lie still on your back and breathe for ~30-40 minutes under a clear Perspex hood to measure your resting metabolic rate. To accurately interpret the results, you will be asked to provide a urine sample on the day of your study investigation visit and collect urine for a 24-hour period. |
| **Vascular ultrasound** | Flow-mediated dilation is a test that measures your vascular health using an ultrasound machine. For the test, a blood pressure cuff will be placed around your upper arm, and you will be asked to lay on your back. The cuff will inflate and tighten for ~5 minutes and then deflate. Ultrasound images will be taken throughout the test. |
| **Bone density scan** | A bone density scan of your spine will tell us about your bone health. During the scan, you will be asked to lie on your back with your legs resting on a foam block. The scan will only take a few minutes and is completed at the same time as the detailed body composition scan. |
| **Near-infrared spectroscopy** | Near-infrared spectroscopy (NIRS) may also be performed at rest and during exercise. NIRS involves placing a device that emits non-harmful light on your muscle. This device can tell us information about the oxygenation of your muscles. |

## Exercise Training

If you consent to take part in this study and are eligible after baseline testing, you will be randomly allocated into one of two groups. You will **NOT** be able to choose which group you are in. Below is a description of what is involved based on which group you are allocated to:

### Telehealth Exercise Training Group

If you are allocated to the telehealth exercise training group, you will be asked to participate in a supervised moderate-to-vigorous intensity exercise training program delivered via an online platform (e.g., Zoom). You will be asked to participate in online small group resistance exercise sessions three times a week for 4-months. The online supervised sessions will take approximately 45-60 minutes. The exercise sessions will involve a variety of exercises using your body weight or a GymStick^TM^ (which will be provided to you). You will also be asked to perform ~30 minutes of aerobic exercise a week (e.g., walking or cycling) independently. You will receive a complimentary gym membership to facilitate your aerobic exercise training.

After the 4-month online exercise training period, there will be an 8-month follow-up period. You will continue to receive a free gym membership courtesy of Fitness First or Goodlife Health Clubs (WA study participants) for the remaining 8-month follow-up period. During the 8-month follow-up period, you will be asked to perform exercise independently, either at home or at the gym, with remote support via phone or email by research staff. You will also be asked to keep a log to record information regarding your exercise training.

Research staff will contact you once every month during your 4-month supervised exercise training period to record information about your exercise training. After the 4-month supervised period, study staff will contact you fortnightly for the initial 2-months and then monthly for the remaining 6-months of the study to provide remote support and record information about your exercise training.

### Waitlist Control Group

If you are allocated to the control group, you will participate in the three study investigation visits (i.e., at baseline testing, 4-months and 12-months). During the 12-month period, you will be asked to continue with your usual treatment and management plan as advised by your cardiologist and medical team. The study team will contact you once a month throughout the 12-month study period for a “health check”.

After your final testing session at 12-months, you will be offered a 4-month supervised moderate-to-vigorous intensity telehealth exercise program delivered via an online platform (e.g., Zoom). In addition, you will also receive a complimentary 12-month gym membership courtesy of Fitness First or Goodlife Health Clubs (WA study participants) after you complete your final testing session.

## Services Australia Data Linkage

The study also wishes to assess if there is any change to your healthcare expenditure prior, during and after your participation in the study, information for which is available via Services Australia. Whether or not you wish for Services Australia to provide this data to us is completely up to you, and your choice will not impact your care or relationship with the hospital or university or study staff. If you choose to link this data, you will be asked to sign a separate consent form authorising the study to access your Services Australia information, see the separate Services Australia Participant Information Document and Participant Consent Form, confirming your consent to provide this data to the study.

Services Australia is not involved in this research other than to provide the information that you have consented to the release of, should you decide to participate in this study. Services Australia has confirmed that this research and any associated documents have received approval from a Human Research Ethics Committee (HREC) that is registered with and operates within guidelines set out by the National Health and Medical Research Council (NHMRC).

# What are the possible benefits?

By participating in this study, you will receive some form of supervised exercise training. In addition, you will gain established health benefits from participating in regular exercise training. The study will provide you and your cardiologist with an opportunity to receive detailed information regarding your health, which may assist your doctor in managing your care in the future. If any abnormalities are found in your testing results, we will liaise with your usual physician to help plan your ongoing care. By participating in this study, you will also help us to understand the effects of exercise training in people living with congenital heart disease.

# What are the possible risks?

Before you are enrolled in this study, with your permission, we will review your medical record and seek approval from your cardiologist about your safety to participate in the study. If we identify any issues (such as uncontrolled heart rhythm problems) that should be optimised before you start an exercise training program for you to exercise safely, we will not enrol you in the study.

All medical procedures - whether for diagnosis or treatment, routine or experimental – involve some risk of injury. In addition, there may be risks associated with this study that are presently unknown and unforeseeable. Despite all precautions, you might develop medical complications from participating in this study.

The tests involved are all very safe and have minimal risk involved, however, it is important to outline the rare but possible risks of each one:

- During exercise training and testing, there may be a small risk of musculoskeletal injury (e.g. muscle strains) as well as abnormal heart rhythm or blood pressure response. To minimise the chance of these events occurring, exercise training sessions will be supervised by an exercise professional and start at low intensities with gradual progression and we will be carefully assessing the information we collect during the initial assessment and exercise tests to help us determine safety to exercise. With your permission, we will also check that your treating cardiologist agrees that you are safe to participate in the study. You should notify the research study team and/or the supervising exercise professional if you experience symptoms including chest pain, dizziness, syncope (fainting), severe breathlessness during low levels of exercise or the new onset of palpitations.
- The body composition scan (DXA) used in this research study involves exposure to a very small amount of radiation. As part of everyday living, everyone is exposed to naturally occurring background radiation and receives a dose of about 2 millisieverts (mSv) each year. The effective dose from this study is about 0.04 mSv. At this dose level, no harmful effects of radiation have been demonstrated as any effect is too small to measure. This risk is believed to be minimal.
- Blood tests have a very small risk of infection and bleeding. This is the same risk as for any blood test that you might have had previously.

# Do I have to take part in the study?

Participation in the study is voluntary. You do not have to take part in the study if you do not wish to. If you decide you want to take part in the study and later change your mind, you are free to withdraw at any stage. Your decision to take part or not will not in any way affect your usual monitoring and treatment for this condition.

# What if I want to withdraw from the study?

If you decide to withdraw from the study, please notify a member of the study team. If you decide to withdraw, the researchers would like to keep any personal and health information that has been collected. If you do not want them to do this, please let us know. This will help to ensure the results of the study can be measured properly.

# How will I be informed of the results of the study?

You will be informed of test results after each one is carried out. If there is a reason to start any new medications or to modify treatment, this will be discussed with you after the test results have been reviewed and your usual doctors will be notified so that they can initiate treatment. Once the study has been completed, a summary of the results will be available from the investigator on request.

# What else do I need to know?

## What will happen to the information collected about me?

Any information obtained in connection with this study that can identify you will remain confidential and will only be used for the purposes of this study. It will only be disclosed with your permission, except as required by law.

Information about you may be obtained from your health records held at this, and other, health services for the purposes of this study. By signing the Participant Consent Form, you authorise the release of, or access to, this confidential information to the relevant study personnel.

In any publication and/or presentation, data /information about you will be provided in a manner that cannot identify you, except with your permission. No identifying material will be used in any reports of this study.

Records for the study will be kept in a secure filing cabinet in a secure office and on a secure online database (Research Electronic Data Capture (REDCap). This is a secure, web-based, non-commercial, data management tool designed for research purposes - hosted and backed up on the Sydney Local Health District (SLHD) servers daily. Only designated study researchers will have access to this secure data.

The physical record of information pertaining to you will be retained for 15 years after which they will be disposed of using the SLHD confidential waste disposal service. Your blood samples will be stored and analysed at the testing centre facilities **[NAME OF HOSPITAL OR UNIVERSITY]** during the study and disposed of consistent with centre-specific protocols upon study completion. Electronic/digital records relating to the study will be kept indefinitely.

Your de-identified data may be shared with other local or international collaborators and used for future research purposes; however, Human Research Ethics Committee (HREC) approval will be sought before any future use of the data. You can indicate your agreement to this on the Participant Consent Form.

## How can I access my information?

In accordance with relevant Australian and/or NSW privacy and other relevant laws, you have the right to access information collected and stored by the researchers about you. You also have the right to request that any information with which you disagree be corrected. Please contact one of the researchers named at the end of this document if you would like to access your information.

## What happens if I am injured as a result of participating in this study?

If you suffer any injuries or complications as a result of this study, you should contact the study doctor as soon as possible, who will assist you in arranging appropriate medical treatment. If you are eligible for Medicare, you can receive any medical treatment required to treat the injury or complication, free of charge, as a public patient in any Australian public hospital.

In addition, you may have a right to take legal action to obtain compensation for any injuries or complications resulting from the study. Compensation may be available if your injury or complication is sufficiently serious and is caused by unsafe drugs or equipment, or by the negligence of one of the parties involved in the study (for example, the researcher, the hospital, or the treating doctor). You do not give up any legal rights to compensation by participating in this study.

## Is this study approved?

The study has been approved by the RPAH Zone Ethics Review Committee. Any person with concerns or complaints about the conduct of this study should contact the Executive Officer on (02) 9515 7176 and quote protocol number X21-0224.

The conduct of this study at the **[NAME OF HOSPITAL OR UNIVERSITY]** has been authorised by the [name of Local Health District]. Any person with concerns or complaints about the conduct of this study may also contact the Research Governance Officer **[OR OTHER OFFICER]** on **[TELEPHONE NUMBER]** and quote protocol number **[INSERT LOCAL PROTOCOL NUMBER]**.

# Who can I contact?

If you want any further information concerning this study or if you have any medical problems which may be related to their involvement in the study (i.e., any side effects), you can contact the following study investigator:

**[INSERT APPROPRIATE STUDY INVESTIGATOR DETAILS]**

This information sheet is for you to keep.

#
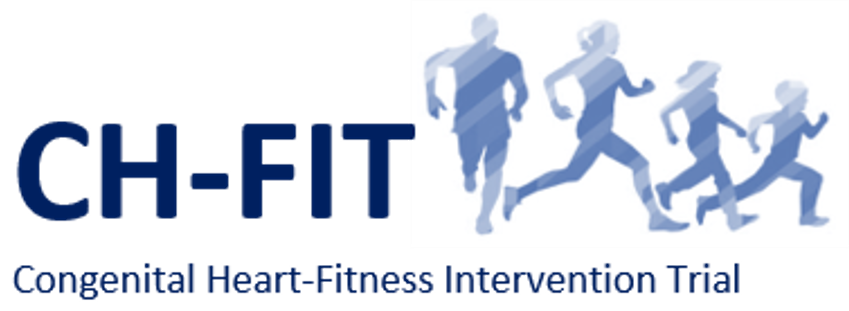
Participant Consent Form

I,_____________________________________________________________________  *[full name]*

Of_____________________________________________________________________ *[address]*

have read and understood the Participant Information Sheet on the above named research study, and have discussed the study with ____________________________________________. __________________ _______ *[investigator responsible for conducting informed consent]*

- I have been made aware of the procedures involved in the study, including any known or expected inconvenience, risk, discomfort or potential side effect and of their implications as far as they are currently known by the researchers.
- I understand that my participation in this study will allow the researchers and others, as described in the Information for Participants, to have access to my medical record, and I agree to this.
- I would like to receive a copy of the cardiac MRI results when they become available. My email address is:

|  |
| --- |
|  |

- I understand that my de-identified data may be used for future research and I agree to this.
- I would like to receive a copy of the study results when they become available.
- I understand that, during this study, my medical records may be accessed by regulatory authorities or by the Ethics Committee approving the research to verify results and determine that the study is being carried out correctly.
- I understand that the SLHD software license for REDCap (Research Electronic Data Capture) will be used to manage the collection and storage of my research data.
- I have had an opportunity to ask questions and I am satisfied with the answers I have received.
- I freely choose to participate in this study and understand that I can withdraw at any time.
- I also understand that the research study is strictly confidential.
- I hereby agree to participate in this research study.
- I consent to the storage and use of my information collected from me for use, as described in the relevant section of the Participant Information Sheet, for:

-This specific research project

-Other research that is closely related to this research project

-Any other research

- I consent to the future use of any data/samples I provide for research purposes. I understand that before they can use any data I provide, they must seek additional ethics approval.

YES  NO

- I consent for other research collaborators to use any data/samples I provide for future research purposes. I understand that before they can use my data, they must seek additional ethics approval.

YES  NO

| **Participant Name:** |  |  |
| --- | --- | --- |

| **Participant Signature:** |  | **Date:** | | | |  |
| --- | --- | --- | --- | --- | --- | --- |
| **Name of Witness:** |  | | | | | |
| **Signature of Witness:** |  | | | **Date:** |  | |
| **Name of Person conducting  informed consent:** |  | | | | | |
| **Signature of Person conducting  informed consent:** |  | | **Date:** | |  | |

# Withdrawal of Consent Form

SITE: <<INSERT NAME>>

**Study title**: Optimising Exercise Prescription and Delivery in Congenital Heart Disease – The Congenital Heart Fitness Intervention Trial: CH-FIT

I hereby wish to WITHDRAW from the study named above. I understand that such a withdrawal WILL NOT jeopardise my treatment or relationship with the hospital or university.

| **Participant Name:** |  |
| --- | --- |
| **Participant Signature:** |  |
| **Date:** |  |
