# Supplementary material for: The effects of telerehabilitation in adults with complex biventricular congenital heart conditions: protocol for a multi-centre, randomised controlled trial—CH-FIT
Source: Trials. 2024 Apr 5;25:239. doi: 10.1186/s13063-024-08019-7 (PMC10998335; doi:10.1186/s13063-024-08019-7)
Supplement: Supplementary file 3 — Supplementary Material 3. [file 13063_2024_8019_MOESM3_ESM.pdf]

Trial Review

To achieve prospective registration, we recommend submitting your trial for registration at the same time as ethics submission.

Updating a registered trial?

New videos with general tips for updating, and how to update recruitment status (Step 7) are now available!

The safety and scientific validity of this study is the responsibility of the study sponsor and investigators. Listing a study does not mean it has been endorsed by the ANZCTR. Before participating in a study, talk to your health care provider and refer to this information for consumers

< BACK

Trial registered on ANZCTR

**Registration number**	ACTRN12622000050752
**Ethics application status**	Approved
**Date submitted**	20/10/2021
**Date registered**	17/01/2022
**Date last updated**	17/01/2022
**Date data sharing statement initially provided**	17/01/2022
**Type of registration**	Prospectively registered

Titles & IDs

**Public title**	The Effect of Exercise Prescription and Delivery in Congenital Heart Disease – The Congenital Heart Fitness Intervention Trial: CH-FIT
**Scientific title**	The Effect of Exercise Prescription and Delivery in Congenital Heart Disease – The Congenital Heart Fitness Intervention Trial: CH-FIT
**Secondary ID [1]**	Medical Research Future Fund (ARGCHDG000016)
**Secondary ID [2]**	National Heart Foundation of Australia Vanguard Grant (102277)
**Universal Trial Number (UTN)**	
**Trial acronym**	CH-FIT
**Linked study record**	

Health condition

Health condition(s) or problem(s) studied:

Congenital Heart Disease

Fontan circulation

Tetralogy of Fallot

Transposition of the Great Arteries

Hypoplastic Left Heart Syndrome

Tricuspid Atresia

**Condition category**	**Condition code**

Cardiovascular	Other cardiovascular diseases
Physical Medicine / Rehabilitation	Other physical medicine / rehabilitation

Intervention/exposure

Study type
Interventional

Description of intervention(s) / exposure
Eligible participants will be randomised to either a supervised (or partially) physical activity and exercise program of moderate to vigorous intensity, followed by an 8-month maintenance period; or usual care (control) group. Throughout the intervention period, participants in the intervention groups will also receive education to promote a healthy lifestyle. In the intervention groups, a 5-10 minute warm-up and cool down will be performed before and after each session. Adherence will be monitored by recording session attendance. Sessions in the 4-month supervised (or partially supervised) phase will be delivered by exercise professionals (e.g., exercise physiologists, physiotherapists) in small groups.

Adolescents and adults with biventricular congenital heart disease will be randomly allocated to a telehealth exercise training group or usual care group; Fontan participants may also be allocated to a traditional exercise training group. Children will be randomly allocated to a physical activity and exercise training group or usual care group.

The exercise intervention in the traditional exercise group will involve supervised aerobic (e.g., cycling) and resistance exercise (e.g., leg press, chest press) training 3 times a week for 4 months. Sessions will be ~60-75 minutes and supervised by exercise professionals. Aerobic exercise will commence at 40%-50% of heart rate reserve (HRR) and progress to 70%-80% HRR, as tolerated. The resistance exercise component will involve 3 sets of 8-12 repetitions at 60% of one-repetition maximum (1RM) and progressed to 70% 1RM.

The telehealth exercise training group will perform supervised resistance exercise 3 times a week for 4-months on Zoom. Each supervised resistance training session will be ~45-60 minutes in duration. Participants will perform 3 sets of 8-12 repetitions of various exercises using bodyweight or a Gymstick. Resistance exercise intensity and progression will be monitored by rating of perceived exertion on the OMNI scale. Participants in the telehealth exercise training group will also be asked to perform 20 minutes of aerobic exercise training (e.g., walking) independently 3 times a week for 4-months. Aerobic exercise intensity will be monitored by HRR and is consistent with the traditional exercise training group.

Children allocated to the intervention group will participate in a 4-month supervised physical activity and exercise training program. Participants will attend a community or fitness facility once a week for ~90 minutes to perform an interval exercise circuit, practice foundational movement skills and engage in physically active games. The average target exercise intensity for the exercise circuit will be 40%-50% HRR initially and will progress to 70%-80% HRR, as tolerated. In addition, children will be provided with tasks to complete at home (e.g., calf raises) that supplements the physical activity program and promotes a healthy lifestyle.

During the 8-month maintenance phase, adolescent and adult participants allocated in the exercise intervention groups will be encouraged to continue exercise training independently at least 2 times a week. Children will be encouraged to be physically active and join sports clubs. Follow-up phone calls will be conducted monthly to facilitate ongoing physical activity participation.

Participants in the usual care group will continue with routine clinical care and attend testing visits at baseline, 4-months, and 12-months.

Intervention code [1]
Treatment: Other

Intervention code [2]
Lifestyle

Intervention code [3]
Rehabilitation

Comparator / control treatment
Participants allocated to the control group will continue with routine clinical care as directed by their medical team.

Control group
Active

Outcomes

Primary outcome [1]
Change in peak oxygen uptake assessed by cardiopulmonary exercise testing

Timepoint [1]
Baseline, 4 months (primary timepoint) and 12 months after the usual care period or starting the exercise program (i.e., 4-months and 12-months from baseline testing for each group).

Secondary outcome [1]
Changes in cardiopulmonary exercise testing measures analysed by a metabolic cart

Timepoint [1]
Baseline, 4 months (primary timepoint) and 12 months after the usual care period or starting the exercise program (i.e., 4-months and 12-months from baseline testing for each group).

Secondary outcome [2]
Changes in respiratory muscle function (only in Fontan participants) assessed by pulmonary function testing instruments (via maximal static inspiratory and expiratory pressures).

Timepoint [2]
Baseline, 4 months (primary timepoint) and 12 months after the usual care period or starting the exercise program (i.e., 4-months and 12-months from baseline testing for each group).

Secondary outcome [3]
Changes in body composition measured by dual-energy x-ray absorptiometry

Timepoint [3]
Baseline, 4 months (primary timepoint) and 12 months after the usual care period or starting the exercise program (i.e., 4-months and 12-months from baseline testing for each group).

**Secondary outcome [4]**	Changes in liver stiffness measure by liver elastography (only in Fontan participants)
Timepoint [4]	Baseline, 4 months (primary timepoint) and 12 months after the usual care period or starting the exercise program (i.e., 4-months and 12-months from baseline testing for each group).
**Secondary outcome [5]**	Changes in neurocognitive function assessed using Cogstate
Timepoint [5]	Baseline, 4 months (primary timepoint) and 12 months after the usual care period or starting the exercise program (i.e., 4-months and 12-months from baseline testing for each group).
**Secondary outcome [6]**	Changes in near-infrared spectroscopy measures including deoxy hemoglobin, oxyhemoglobin, and skeletal muscle oxidative capacity (composite outcome).
Timepoint [6]	Baseline, 4 months (primary timepoint) and 12 months after the usual care period or starting the exercise program (i.e., 4-months and 12-months from baseline testing for each group).
**Secondary outcome [7]**	Changes in physical activity levels measured by accelerometers
Timepoint [7]	Baseline, 4 months (primary timepoint) and 12 months after the usual care period or starting the exercise program (i.e., 4-months and 12-months from baseline testing for each group).
**Secondary outcome [8]**	Changes in vascular function assessed by flow-mediated dilation (only in Fontan participants)
Timepoint [8]	Baseline, 4 months (primary timepoint) and 12 months after the usual care period or starting the exercise program (i.e., 4-months and 12-months from baseline testing for each group).
**Secondary outcome [9]**	Change in NT-proBNP assessed using standard (serum) assays.
Timepoint [9]	Baseline, 4 months (primary timepoint) and 12 months after the usual care period or starting the exercise program (i.e., 4-months and 12-months from baseline testing for each group).
**Secondary outcome [10]**	Changes in cardiac function assessed by transthoracic echocardiography (only in Fontan participants)
Timepoint [10]	Baseline, 4 months (primary timepoint) and 12 months after the usual care period or starting the exercise program (i.e., 4-months and 12-months from baseline testing for each group).
**Secondary outcome [11]**	Changes in cardiac function assessed by resting and exercise cardiac MRI (only in Fontan participants)
Timepoint [11]	Baseline, 4 months (primary timepoint) and 12 months after the usual care period or starting the exercise program (i.e., 4-months and 12-months from baseline testing for each group).
**Secondary outcome [12]**	Changes in health-related quality of life assessed by PedsQL
Timepoint [12]	Baseline, 4 months (primary timepoint) and 12 months after the usual care period or starting the exercise program (i.e., 4-months and 12-months from baseline testing for each group).
**Secondary outcome [13]**	Changes in health economics and patient costs assessed by data-linkage to medicare, CHU-9D and EQ5D.
Timepoint [13]	Baseline, 4 months (primary timepoint) and 12 months after the usual care period or starting the exercise program (i.e., 4-months and 12-months from baseline testing for each group).
**Secondary outcome [14]**	Changes lung function (only in Fontan participants) assessed by pulmonary function testing instruments (via spirometry and body plethysmography).
Timepoint [14]	Baseline, 4 months (primary timepoint) and 12 months after the usual care period or starting the exercise program (i.e., 4-months and 12-months from baseline testing for each group).
**Secondary outcome [15]**	Changes in handgrip strength (in children, adolescents, and adults) assessed by a hand-grip dynamometer.
Timepoint [15]	Baseline, 4 months (primary timepoint) and 12 months after the usual care period or starting the exercise program (i.e., 4-months and 12-months from baseline testing for each group).
**Secondary outcome [16]**	Changes in one-repetition maximum assessed by one-repetition maximum testing (in adolescents and adults).
Timepoint [16]	Baseline, 4 months (primary timepoint) and 12 months after the usual care period or starting the exercise program (i.e., 4-months and 12-months from baseline testing for each group).
**Secondary outcome [17]**	Changes in and muscular endurance assessed by musculoskeletal endurance testing (in adolescents and adults).
Timepoint [17]	Baseline, 4 months (primary timepoint) and 12 months after the usual care period or starting the exercise program (i.e., 4-months and 12-months from baseline testing for each group).
**Secondary outcome [18]**	Changes in the number of push-ups performed (in children).
Timepoint [18]	Baseline, 4 months (primary timepoint) and 12 months after the usual care period or starting the exercise program (i.e., 4-months and 12-months from baseline testing for each group).
**Secondary outcome [19]**	Changes in standing long jump distance (in children).
Timepoint [19]	Baseline, 4 months (primary timepoint) and 12 months after the usual care period or starting the exercise program (i.e., 4-months and 12-months from baseline testing for each group).
**Secondary outcome [20]**	Changes in dietary and nutrition intake assessed by the ASA24.
Timepoint [20]	Baseline, 4 months (primary timepoint) and 12 months after the usual care period or starting the exercise program (i.e., 4-months and 12-months from baseline testing for each group).
**Secondary outcome [21]**	Changes in malnutrition assessed by the SGA or SGNA
Timepoint [21]	Baseline, 4 months (primary timepoint) and 12 months after the usual care period or starting the exercise program (i.e., 4-months and 12-months from baseline testing for each group).
**Secondary outcome [22]**	Changes in energy expenditure assessed by and indirect calorimetry.

Timepoint [22]	Baseline, 4 months (primary timepoint) and 12 months after the usual care period or starting the exercise program (i.e., 4-months and 12-months from baseline testing for each group).
**Secondary outcome [23]**	Changes in gastrointestinal symptoms assessed by the GSRS.
Timepoint [23]	Baseline, 4 months (primary timepoint) and 12 months after the usual care period or starting the exercise program (i.e., 4-months and 12-months from baseline testing for each group).

Eligibility

**Key inclusion criteria**	-Aged 10 to 55 years<br>-People with congenital heart disease of moderate or great complexity<br>-At least 6 months post surgical repair<br>-Medically stable and on stable therapy for at least 3 months
**Minimum age**	10 Years
**Maximum age**	55 Years
**Sex**	Both males and females
**Can healthy volunteers participate?**	No
**Key exclusion criteria**	-Physiological stage D<br>-Planned intervention within 2 years<br>-Mental or physical disability that prevents participation in exercise training<br>-Current or actively planned pregnancy within one year<br>-Uncontrolled (systemic) hypertension at rest of exercise<br>-Clinically unstable or recent major change in therapy within 3 months<br>-COVID-19 unvaccinated people despite being eligible according to ATAGI<br>-People with unreliable internet connection<br>-People currently participating in more than 30 minutes of sports or exercise training three times a week

Study design

**Purpose of the study**	Treatment
**Allocation to intervention**	Randomised controlled trial
**Procedure for enrolling a subject and allocating the treatment (allocation concealment procedures)**	
**Methods used to generate the sequence in which subjects will be randomised (sequence generation)**	
**Masking / blinding**	
**Who is / are masked / blinded?**	
**Intervention assignment**	
**Other design features**	
**Phase**	Not Applicable
**Type of endpoint/s**	Safety/efficacy
**Statistical methods / analysis**	

Recruitment

**Recruitment status**	Not yet recruiting

Date of first participant enrolment

Anticipated 1/03/2022 **Actual**

Date of last participant enrolment

Anticipated **Actual**

Date of last data collection

Anticipated **Actual**

Sample size

Target 370 **Accrual to date** **Final**

Recruitment in Australia

**Recruitment state(s)**	NSW,QLD,WA,VIC
**Recruitment hospital [1]**	Royal Prince Alfred Hospital - Camperdown
**Recruitment hospital [2]**	The Prince Charles Hospital - Chermside
**Recruitment hospital [3]**	Fiona Stanley Hospital - Murdoch
**Recruitment hospital [4]**	The Royal Childrens Hospital - Parkville
**Recruitment hospital [5]**	Perth Children's Hospital - Nedlands
**Recruitment hospital [6]**	The Children's Hospital at Westmead - Westmead
**Recruitment hospital [7]**	Queensland Children's Hospital - South Brisbane
**Recruitment hospital [8]**	Royal Melbourne Hospital - City campus - Parkville
**Recruitment postcode(s) [1]**	2050 - Camperdown
**Recruitment postcode(s) [2]**	3050 - Parkville
**Recruitment postcode(s) [3]**	4032 - Chermside
**Recruitment postcode(s) [4]**	6150 - Murdoch
**Recruitment postcode(s) [5]**	6009 - Nedlands
**Recruitment postcode(s) [6]**	2145 - Westmead
**Recruitment postcode(s) [7]**	4101 - South Brisbane
**Recruitment postcode(s) [8]**	3050 - Royal Melbourne Hospital

Funding & Sponsors

**Funding source category [1]**	Government body
**Name [1]**	National Health & Medical Research Council, the Medical Research Future Fund (MRFF) – Congenital Heart Disease
**Address [1]**	16 Marcus Clarke St, Canberra ACT 2601
**Country [1]**	Australia
**Funding source category [2]**	Charities/Societies/Foundations
**Name [2]**	Additional Ventures
**Address [2]**	314 Lytton Ave Suite 200, Palo Alto, CA 94301
**Country [2]**	United States of America
**Funding source category [3]**	Charities/Societies/Foundations
**Name [3]**	Vanguard grants - Heart Foundation, Australia
**Address [3]**	80 William St, Woolloomooloo NSW 2011
**Country [3]**	Australia
**Primary sponsor type**	University
**Name**	University of Sydney
**Address**	Camperdown NSW 2006
**Country**	Australia
**Secondary sponsor category [1]**	None
**Name [1]**	
**Address [1]**	
**Country [1]**	

Ethics approval

**Ethics application status**	Approved
**Ethics committee name [1]**	Sydney Local Health District Human Research Ethics Committee
**Ethics committee address [1]**	Level 11, KGV Building Missenden Road CAMPERDOWN NSW 2050
**Ethics committee country [1]**	Australia
**Date submitted for ethics approval [1]**	25/06/2021
**Approval date [1]**	03/09/2021
**Ethics approval number [1]**	Protocol no. X21-0224 & 2021/ETH01181

Summary

Brief summary

Congenital heart disease (CHD) is the most common congenital disorder in newborns, with an estimated 2400 Australian babies born with CHD each year. Despite improved survival due to advances in surgical techniques and medical care, premature morbidity and mortality are common. In this multi-centre randomised controlled trial, eligible participants with CHD will be allocated to either a 4-month face-to-face or telehealth moderate-to-vigorous physical activity and exercise training program or a usual care (control) group. Participants allocated in the intervention groups will also engage in an 8-month maintenance phase. Detailed assessment of exercise capacity, body composition, vascular function, physical activity levels, liver stiffness, cardiac function, quality of life, cognitive function, lung function, dietary and nutritional status, peripheral venous pressure, and musculoskeletal fitness will be performed. Testing and assessments will be performed at baseline, 4-months, and 12-months.

Trial website

Public notes

Contacts

Principal investigator

Name Dr Rachel Cordina

Address Suite 303 - RPAH Medical Centre 100 Carillon Ave Newtown NSW 2042

Country Australia

Phone +61 2 9517 4828

Fax

Email rachael.cordina@sydney.edu.au

Contact person for public queries

Name Mr Derek Tran

Address ROYAL PRINCE ALFRED HOSPITAL
Cardiovascular Research Precinct | Department of Cardiology
Level 6 Missenden Road, Camperdown NSW 2050

Country Australia

Phone +61 2 8627 8644

Fax

Email derek.tran@sydney.edu.au

Contact person for scientific queries

Name Mr Derek Tran

Address ROYAL PRINCE ALFRED HOSPITAL
Cardiovascular Research Precinct | Department of Cardiology
Level 6 Missenden Road, Camperdown NSW 2050

Country Australia

Phone +61 2 8627 8644

Fax

Email derek.tran@sydney.edu.au

Data sharing statement

Will individual participant data (IPD) for this trial be available (including data dictionaries)? No

No/undecided IPD sharing reason/comment

What supporting documents are/will be available? No other documents available

Summary results

No Results

< BACK

ANZCTR

Home
About us
Statistics
Useful links
News
Contact
Privacy
Terms and conditions

Register a trial

Create account
Login
How to register a trial
How to update a trial
Data item definitions
Hints and tips
FAQs

Search for a trial

Find a trial
How to search
How to get involved

Major funders

Privacy | Disclaimer
Web design by G Squared

Copyright © Australian New Zealand Clinical Trials Registry. All rights reserved.
